# Feeding Animals

**Published:** 1883-07

**Authors:** 


### REVIEWS.

FEEDING ANIMALS: A PRACTICAL WORK UPON THE LAWS OF ANIMAL GROWTH, ESPECIALLY APPLIED TO THE REARING AND FEEDING OF HORSES, CATTLE, DAIRY COWS, SHEEP AND SWINE. By Elliott W. Stewart. one of the editors of the *National Live Stock Journal*; late non-resident Professor of the Principles of Agriculture in Cornell University. With Illustrations. Sold by the Author. Price, $2. Lake View, Erie Co., New York.

The above is the title of a work, the result of thirty years of enlightened experience, One of the rare things to be desired is a book compiled from such a source, and is to be preferred to others containing many theories advanced after thirty minutes of profound reflexion.

A careful perusal of Mr. Stewart's work stamps it emphatically as a practical, common sense production of long and earnest painstaking labor.

Mr. Stewart sensibly says in the preface to his book: "The author has has not ventured into the discussion of veterinary remedies." But then, he throws cold water on his practical labors by adding: "Contenting himself with the description of a few simple water remedies, endeavoring to impress the reader with the necessity of preventing diseases rather than that of curing them." This is the only theory Mr. Stewart indulges in, and as his intentions are prompted by a kind heart, and as he is a layman, the veterinarians can afford to generously excuse him. It would have been far wiser in the author to have referred to Mr. Jenning's or some other catalogues of veterinary works for information on the subject. We leave this matter to remark that the *analytical index* is studiously and faithfully compiled from the pages of the word. It is a fair sample of Mr. Stewart's painstaking methods of labor. "Feeding Animals" is replete with food for the careful student. The railroad companies, omnibus companies, and all companies using horses should have a copy of Mr. Stewart's work to guide and instruct them to properly feed their hard worked horses. Every stable in the country should have "Feeding Animals," if no other book. If any veterinarian will handle the subject of treatment and care of horses, or other animals as practically as Mr. Stewart has done we will willingly commend his labor in this Journal. We sincerely recommend "Feeding Animals" to the general public.

